# Supplementary material for: Collaborative intelligence in AI: Evaluating the performance of a council of AIs on the USMLE
Source: PLOS Digit Health. 2025 Oct 9;4(10):e0000787. doi: 10.1371/journal.pdig.0000787 (PMC12510544; doi:10.1371/journal.pdig.0000787)
Supplement: S2 File — (ZIP) [file pdig.0000787.s002.zip › S2 Data Raw data files containing unprocessed question inputs and LLM outputs including transcripts of deliberations/STEP3_Question-95-1_Type-MC-NJ.docx]

**Facilitator** **[Round 1]:** A 26-year-old man, who is admitted to the hospital to undergo cholecystectomy, bleeds excessively during the procedure and then develops a large wound hematoma. Medical history discloses that he has always tended to bruise excessively with trauma. His wounds oozed blood for 3 days following a dental extraction, which was his only previous surgical procedure. He says that his maternal grandfather and uncle were ""bleeders."" Initial coagulation studies show a normal platelet count, bleeding time, and prothrombin time. The partial thromboplastin time is moderately prolonged. Which of the following is the most appropriate statement to this patient and his wife regarding their children? (A) There is no evidence of a familial coagulation defect (B) Half of their daughters will have a clinically evident coagulation disorder (C) The sons of their daughters will be at risk for a clinically evident coagulation disorder (D) Their sons will be at risk for a clinically evident coagulation disorder (E) Their sons and daughters will be at risk for a significant coagulation disorder

**AI_E:** (1) RESTATING THE QUESTION: This case involves a 26-year-old man who tends to bleed excessively when injured or undergoing surgery, and this problem seems to run in his maternal family line. His blood test results were normal in most respects, but showed an increased partial thromboplastin time. The question is asking what will be the most suitable advice to this patient and his wife in terms of the genetic risk for their children to have similar coagulation issues. (2) PLANNING: On reviewing the findings, we see this patient presents with a prolonged partial thromboplastin time which indicates that the intrinsic clotting pathway is affected, and it seems to be familial. This pattern suggests an X-linked recessive pattern of inheritance likely, such as hemophilia. Hemophilia is a group of disorders characterized by an inability to form clots due to a deficiency in certain clotting factors. It's assumed that the patient and his wife will consider their children's potential risk of inheriting this condition. (3) CLINICAL REASONING: From a genetic perspective, if the father carries an X-linked recessive trait, the sons will be unaffected, as they receive their X chromosome from their mother. On the other hand, all their daughters will be carriers because they inherit his affected X chromosome. However, until they conceive sons, these carrier daughters generally won't display symptoms of the disorder. Considering the maternal uncle and grandfather were also affected, it reinforces that this disorder probably follows an X-linked recessive pattern. (4) CONCLUDING: Therefore, the children of this couple are at risk of inheriting this coagulation defect, but only the sons of their daughters will likely display symptoms. That's because their daughters (i.e., grandsons from the patient’s side) have a 50% chance of inheriting the defective gene carried by their mother. (5) RESPONSE: Thus, the most appropriate statement to this patient and his wife regarding their children would be: the sons of their daughters will be at risk for a clinically evident coagulation disorder (Option C).

**AI_B:** The main question we need to answer here concerns the risk of the patient's children having a coagulation disorder, that was characterized by excessive bleeding during surgery, prolonged oozing after dental extraction and a familial history of being "bleeders". The disorder appears to be hereditary since the patient mentioned that his maternal grandfather and uncle also had the same issue. The lab results specifically show a prolonged partial thromboplastin time, which suggests an issue with the intrinsic or common pathway of coagulation. So, the first question is: What coagulation disorder could cause these symptoms? One interesting point to examine is the fact that the patient's grandfather and uncle also had the condition, but there is no mention of his mother being a "bleeder". This could potentially imply a sex-linked genetic pattern because if it were an autosomal dominant trend, we would probably have seen symptoms in the mother as well. Therefore, this leads to an alternate assumption suggesting the disorder may be X-linked recessive. In the realm of coagulation, one common X-linked recessive disorder is Hemophilia A or B, both characterized by deficiency or dysfunction of factor VIII or IX, respectively, which are part of the intrinsic coagulation pathway. Prolonged partial thromboplastin time is often found in patients with hemophilia since this test measures factor VIII and IX. Moreover, it's worth appreciating the inheritance pattern of X-linked recessive disorders – only males manifest the disease, while females can act as carriers. A son is not at risk to inherit this disease from his father because he inherits his Y chromosome from the father and X from the mother. However, a daughter inherits an X chromosome from each parent, putting her at risk to become a carrier if her father has the disease. In this scenario, the most appropriate statement about their children's risk considering these assumptions would be – "the sons of their daughters will be at risk for a clinically evident coagulation disorder" (C). It doesn't mean that their daughters are guaranteed to inherit the aberrant gene (it will be a 50% possibility), but if they do happen to become carriers, their sons would run a risk of manifesting the condition.

**AI_C:** (1) RESTATING THE QUESTION: The situation presents a 26-year-old male patient displaying signs of a possible clotting disorder, such as excessive bleeding and hematoma formation following surgery. His personal and family medical history points towards a hereditary coagulation defect. Initial coagulation studies showed normal platelet count, bleeding time, and prothrombin time, but moderately prolonged partial thromboplastin time. The query is about which statement would be most appropriate to inform the patient and his wife about the potential risk of their children inheriting this potential disorder. (2) PLANNING: The case information hints the presence of a possible inherited blood coagulation disorder. The inheritance pattern appears to be X-linked due to the male patient's maternal relatives (grandfather and uncle) also having bleeding tendencies. It's important to know that X-linked disorders primarily affect males. X-linked inheritance means that the gene causing the trait or the disorder is located on the X chromosome. Females have two X chromosomes, so even when one has the gene, the normal gene on the other chromosome can often compensate. Males, however, have only one X chromosome, so if they have the gene, they will develop the trait or the disorder. Based on this, the assumption here is that if the patient's disorder is truly an X-linked disorder, his children's risk of manifesting a clinically evident coagulation disorder will depend on their gender. (3) CLINICAL REASONING: To expand on this assumption, if this patient and his wife have children, his daughters would each get one X from the mom (normal) and the X from the patient (with the clotting disorder gene). Thus, they're carriers but may not show symptoms of the disease due to the compensation by the normal X chromosome. The sons, on the other hand, would receive the Y from the patient (which doesn't carry the clotting disorder gene) and an X from the wife (normal), so they don't inherit the disease. However, the daughters have a 50% chance of passing on the affected X chromosome to their children, thus if they have male children (grandsons of the patient), those male children would have a risk of presenting the bleeding disorder. (4) CONCLUDING: For the couple's daughters, they may be carriers of the disorder without any clinical manifestation, while their sons would not inherit the disorder. However, the male offspring (sons) of their daughters would be at risk for a clinically evident coagulation disorder. (5) RESPONSE: The most appropriate statement to this patient and his wife regarding their children would be, (C) The sons of their daughters will be at risk for a clinically evident coagulation disorder.

**AI_A:** RESTATING THE QUESTION: The patient, a 26-year-old man, has shown a pattern of excessive bleeding. This was evidenced during a surgical procedure where he developed a large wound hematoma and his personal medical history reveals a tendency to bruise excessively with trauma. His only other surgical procedure, dental extraction, resulted in prolonged bleeding. He reported a familial history of similar symptoms, his maternal grandfather and uncle were termed "bleeders". Coagulation tests showed normal count of platelets, bleeding time, and prothrombin time, but partial thromboplastin time was long. The question asks what the implications are for this patient's potential children given his pattern of symptoms and the results of his coagulation studies. PLANNING: The basic assumption is that the patient has an inherited bleeding disorder. The evidence supporting this assumption includes the patient's clinical symptoms and family history. However, we should question whether another explanation for his symptoms could exist, and we should also consider the specific type of genetic disorder that may be present, since this will impact the risk to his future children. CLINICAL REASONING: The normal platelet count, bleeding time and prothrombin time, along with a prolonged partial thromboplastin time might suggest a defect in the intrinsic and/or common pathways of coagulation cascade. Combined with the patient's description of his maternal relatives as “bleeders”, these findings are suggestive of an X-linked recessive disorder like hemophilia. Hemophilia is only seen in males, although females can be carriers. Daughters of affected men are obligate carriers of the disorder (since they'll always inherit his affected X chromosome), while sons of affected men will not inherit the disorder (since they inherit the father's Y chromosome). CONCLUDING: Therefore, the bleeding disorder in this case seems to be affecting the intrinsic pathway of coagulation and is likely X-linked recessive as per the familial history. Due to the pattern of inheritance of X-linked recessive disorders, the sons of this patient will not inherit the disorder, but his daughters will become carriers. This means that his daughters' sons (i.e., his grandsons) will have a risk of expressing the disorder. RESPONSE: Hence, the most suitable statement to make to this patient and his wife regarding their future children would be that "(C) The sons of their daughters will be at risk for a clinically evident coagulation disorder".

**AI_D:** (1) RESTATING THE QUESTION: What we're examining here is the way a particular genetic disorder, in this case a bleeding disorder with a moderately prolonged partial thromboplastin time, is likely to be transmitted within a family. The man in question appears to be affected by this disorder, and it has been suggested that his maternal grandfather and uncle had the same issue. We need to identify which of this man's potential future children may inherit this condition. (2) PLANNING: In analyzing the presented clinical scenario, let's consider what information we know: we have a male patient who bleeds excessively with trauma, whose coagulation studies only show a prolonged partial thromboplastin time, and who reports a family history of bleeding issues on his mother's side. The maternal lineage, combined with the specific symptoms and tests, hints at a genetic bleeding disorder, specifically a type of Hemophilia, possibly Hemophilia A or B, which are both X-linked recessive disorders. As a male, our patient has only one X-chromosome, which if affected, would manifest the disease. Continuing under this hypothesis, we can predict some patterns of inheritance for this condition. For X-linked disorders, if a father is affected, none of his sons would inherit the disorder (as sons receive only the Y chromosome from their fathers), but all his daughters will all be carriers (as daughters receive the father's X chromosome). The grandchildren will then have different risks based on whether their parent is an affected male, unaffected male, carrier female, or non-carrier female. (3) CLINICAL REASONING: Given the pattern of this potential X-linked disorder, the patient’s sons, receiving only the Y chromosome from their father, would not be at risk for this coagulation disorder. All daughters, however, will receive the affected X-chromosome from their father, making them carriers of the disorder. As carriers, they will typically not have clinically significant bleeding, but sons of these carriers could inherit the affected X-chromosome, putting them at risk for an evident coagulation disorder. (4) CONCLUDING: Therefore, the best statement to explain to this patient and his wife regarding the possibility of transmission of this potential genetic coagulation disorder towards their children would outline that their daughters could be carriers, but are unlikely to have clinically significant disease, whereas their grandchildren (particularly the sons of their daughters) could be affected by the disorder. (5) RESPONSE: As such, the most appropriate response is option (C) The sons of their daughters will be at risk for a clinically evident coagulation disorder.

**Facilitator** The consensus among the AI responses is that the patient's symptoms, family history, and coagulation studies suggest an inherited bleeding disorder, likely an X-linked recessive disorder such as hemophilia. This is supported by the patient's excessive bleeding, prolonged partial thromboplastin time, and the fact that his maternal grandfather and uncle were also "bleeders". In terms of inheritance, X-linked recessive disorders primarily affect males, as they have only one X chromosome. If a father is affected, none of his sons would inherit the disorder, as they receive the Y chromosome from their father. However, all his daughters will inherit the affected X chromosome, making them carriers of the disorder. As carriers, they typically do not show symptoms of the disease, but their sons (the patient's grandsons) could inherit the affected X chromosome, putting them at risk for the disorder. Therefore, the most appropriate statement to this patient and his wife regarding their future children would be that their daughters could be carriers, but are unlikely to have clinically significant disease, whereas their grandchildren, particularly the sons of their daughters, could be affected by the disorder. Hence, the most suitable response is option (C) The sons of their daughters will be at risk for a clinically evident coagulation disorder.
